# The Role of *Helicobacter pylori* Neutrophil-Activating Protein in the Pathogenesis of *H. pylori* and Beyond: From a Virulence Factor to Therapeutic Targets and Therapeutic Agents

**DOI:** 10.3390/ijms24010091

**Published:** 2022-12-21

**Authors:** Hua-Wen Fu, Yu-Chang Lai

**Affiliations:** 1 Institute of Molecular and Cellular Biology, National Tsing Hua University, Hsinchu 30013, Taiwan; 2 Department of Life Science, National Tsing Hua University, Hsinchu 30013, Taiwan

**Keywords:** *Helicobacter pylori* neutrophil-activating protein, HP-NAP, *H. pylori*, GPCR, TLR2, gastric inflammation, immunomodulation, vaccine, adjuvant, allergy treatment, cancer immunotherapy

## Abstract

*Helicobacter pylori* neutrophil-activating protein (HP-NAP), a major virulence factor of *H. pylori*, plays a role in bacterial protection and host inflammation. HP-NAP activates a variety of innate immune cells, including neutrophils, monocytes, and mast cells, to induce their pro-oxidant and pro-inflammatory activities. This protein also induces T-helper type 1 (Th1) immune response and cytotoxic T lymphocyte (CTL) activity, supporting that HP-NAP is able to promote gastric inflammation by activation of adaptive immune responses. Thus, HP-NAP is a potential therapeutic target for the treatment of *H. pylori*-induced gastric inflammation. The inflammatory responses triggered by HP-NAP are mediated by a PTX-sensitive G protein-coupled receptor and Toll-like receptor 2. Drugs designed to block the interactions between HP-NAP and its receptors could alleviate the inflammation in gastric mucosa caused by *H. pylori* infection. In addition, HP-NAP acts as a promising therapeutic agent for vaccine development, allergy treatment, and cancer immunotherapy. The high antigenicity of HP-NAP makes this protein a component of vaccines against *H. pylori* infection. Due to its immunomodulatory activity to stimulate the Th1-inducing ability of dendritic cells, enhance Th1 immune response and CTL activity, and suppress Th2-mediated allergic responses, HP-NAP could also act as an adjuvant in vaccines, a drug candidate against allergic diseases, and an immunotherapeutic agent for cancer. This review highlights the role of HP-NAP in the pathogenesis of *H. pylori* and the potential for this protein to be a therapeutic target in the treatment of *H. pylori* infection and therapeutic agents against *H. pylori*-associated diseases, allergies, and cancer.

## 1. Introduction

*Helicobacter pylori* (*H. pylori*), a microaerophilic, Gram-negative bacterium first isolated by Barry Marshall and Robin Warren in 1982, has been recognized to be the most common cause of chronic gastritis and peptic ulcer disease [1,2,3]. This bacterium is one of the most successful human pathogens because approximately half of the human population is infected by *H. pylori* [4] The situation is worse in developing countries, in which over 80% of the population might be *H. pylori*-infected [5]. *H. pylori* was classified to be a class I carcinogen by the International Agency for Research on Cancer (IARC) in 1994 [6]. Gastric cancer induced by *H. pylori* accounts for more than 5% of the global cancer burden [7,8]. It is also established that this bacterium attributes to approximately 90% of non-cardia gastric cancer [7]. Due to the prevalence and severity of *H. pylori* infection, it is important to develop strategies for the prevention and treatment of *H. pylori*-associated gastric diseases. To achieve this goal, researchers have identified and characterized the virulence factors of *H. pylori* and investigated their roles in the pathogenesis of *H. pylori*. Some putative virulence factors, such as urease, cytotoxin-associated gene A (CagA), vacuolating cytotoxin gene (VacA), *H. pylori* neutrophil-activation protein (HP-NAP), duodenal ulcer promoting gene (DupA), and blood group antigen-binding adhesion (BabA), have been reported to be associated with the clinical outcomes of *H. pylori* infection [9,10,11].

Among the known virulence factors of *H. pylori*, HP-NAP, first isolated and identified in 1995, was named for its ability to activate neutrophils to produce reactive oxygen species (ROS) and to adhere to endothelial cells [12]. HP-NAP is a spherical protein consisting of 12 identical subunits [13]. Each subunit of the dodecameric HP-NAP is a four-helix bundle protein with a molecular mass close to 17 kDa [13,14]. It belongs to the family of the DNA-binding protein from starved cells (Dps), whose structures closely resemble those of bacterial ferritins [15,16]. Due to its capability of sequestering free iron and binding DNA, HP-NAP protects *H. pylori* from oxidative DNA damage [17,18,19,20]. Such protection contributes much to the survival of *H. pylori*. In addition to its role in bacterial protection, HP-NAP elicits various immune responses to induce gastric inflammation during *H. pylori* infection [21]. This protein is also an important antigen of *H. pylori* and processes immunomodulatory properties [22,23]. As a major virulence factor contributing to *H. pylori*-induced gastric inflammation, HP-NAP is an ideal subject for studying its role in the pathogenesis of *H. pylori* and a potential therapeutic target for *H. pylori*-associated gastric diseases. Furthermore, its unique immune properties make HP-NAP a potential therapeutic agent against *H. pylori* infection, allergic diseases, and cancers.

## 2. HP-NAP as a Virulence Factor of *H. pylori*

One of the earliest known biological activities of HP-NAP is the induction of ROS production by neutrophils [12]. It was later reported that HP-NAP stimulated ROS production by neutrophils and monocytes in a dose-dependent manner [22]. The ROS production induced by HP-NAP is mediated by nicotinamide adenine dinucleotide phosphate (NADPH) oxidase on the plasma membrane of neutrophils, as indicated by the translocation of its components from the cytosol to the membrane [22]. In addition, HP-NAP-induced ROS production is mediated by the signaling pathway involving pertussis toxin (PTX)-sensitive heterotrimeric G proteins, phosphatidylinositol 3-kinases, Src family tyrosine kinases, and a rise in cytosolic calcium [22], suggesting that a PTX-sensitive G protein-coupled receptor (GPCR) participates in this event. However, the GPCR of HP-NAP remains to be identified. The oxidative stress induced by HP-NAP damages the host gastric epithelial cells, while it also kills the invaded *H. pylori*. Interestingly, it has been reported that ROS can promote *H. pylori* to form biofilm for protecting itself from the toxicity caused by oxidative stress [24]. HP-NAP, identified as a *H. pylori* biofilm-associated protein, also participates in the oxidative stress-induced biofilm production of *H. pylori* to confer drug resistance [24,25]. Thus, HP-NAP not only causes oxidative stress in hosts via the induction of ROS production by neutrophils and monocytes but also protects *H. pylori* against the damage caused by oxidative stress through various mechanisms.

The other one of the earliest known biological activities of HP-NAP is the promotion of neutrophils to adhere to endothelial cells [12]. HP-NAP upregulates the expression of β2 integrin by neutrophils and monocytes and induces the conformational change of β2 integrin into a high-affinity state [22,26], supporting a direct role of HP-NAP in the stimulation of neutrophils adhesion to endothelial cells. HP-NAP is a chemotactic factor for neutrophils and monocytes and the chemotactic migration of neutrophils and monocytes toward HP-NAP is dose-dependent [22]. In addition, HP-NAP promotes the transendothelial migration of neutrophils [27]. An in vivo study has further shown that HP-NAP could induce leukocyte adhesion to vessel endothelium tissue as well as leukocyte extravasation, though at a much higher concentration of HP-NAP for the latter [26]. Thus, HP-NAP contributes to the initial recruitment of neutrophils to *H. pylori*-infected gastric mucosa.

Later on, HP-NAP was found to trigger various inflammatory responses via the activation of innate immune cells, including neutrophils, monocytes, and mast cells. HP-NAP induces the secretion of myeloperoxidase by neutrophils [28], indicating that HP-NAP promotes the degranulation of neutrophils. HP-NAP stimulates neutrophils to synthesize and secrete several chemokines, including interleukine-8 (IL-8), macrophage inflammatory protein (MIP)-1α, and MIP-1β [26]. The IL-8 secreted by neutrophils further recruits neutrophils and other immune cells to the site of *H. pylori* infection. It was later found that HP-NAP-induced secretion of IL-8 by neutrophils is mediated by both Toll-like receptor 2 (TLR2) and PTX-sensitive G proteins [29]. In addition to neutrophils, HP-NAP induces monocytes to secrete pro-inflammatory cytokines, such as tumor necrosis factor-α (TNF-α), IL-6, IL-8, IL-12, and IL-23 [30], and mast cells to release IL-6 and histamine [31,32]. Among them, TNF-α also potentiates HP-NAP-induced ROS production by neutrophils [22]. These inflammatory mediators induced by HP-NAP could initiate and modulate inflammation of gastric mucosa during *H. pylori* infection. In addition, HP-NAP stimulates the production of tissue factor and plasminogen activator inhibitor-2 by mononuclear cells [33]. Procoagulant potential and antifibrinolytic activity of mononuclear cells induced by HP-NAP may also contribute to the inflammatory reaction of gastric mucosa caused by *H. pylori* infection. The ability of HP-NAP to elicit innate inflammatory responses supports a pathogenic role of this protein in gastric inflammation during *H. pylori* infection.

HP-NAP induces not only innate immune responses but also adaptive immune responses. HP-NAP stimulates the release of IL-12 and IL-23 by neutrophils and monocytes to promote the polarization of T-helper type 1 (Th1) responses and the differentiation of monocytes toward matured dendritic cells (DCs) [30]. HP-NAP further induces the matured DCs to express major histocompatibility complex (MHC) class II molecules and secrete Th1-polarizing cytokines, such as IL-12 [30,34]. In the IL-12-enriched environment created by HP-NAP, antigen-specific gastric T cell lines produced high amounts of interferon-γ (IFN-γ) and TNF-α and exhibited cytotoxic activity [30], indicating the ability of HP-NAP to promote the Th1 immune response and cytotoxic T lymphocyte (CTL) activity. An in vivo study also shows that HP-NAP-specific T cell clones derived from the gastric mucosa of *H. pylori*-infected patients exhibited a Th1 cell-mediated cytokine profile and cytotoxic activity and expressed TNF-α [30]. Since acute infection with *H. pylori* causes a predominant Th1 response in the gastric antrum of *H. pylori*-infected patients [35,36], these findings support that HP-NAP is a key factor to drive Th1 immune responses during *H. pylor*i infection. The Th1 immune response and CTL activity induced by HP-NAP could also lead to the inflammation and damage of gastric mucosa during *H. pylori* infection.

Although the immune cell responses induced by HP-NAP have been well characterized, it is not exactly known how this protein is released from *H. pylori* and recognized by the immune cells in tissues or in circulation. It has been reported that HP-NAP is an outer membrane protein located at the surface of *H. pylori* to act as an adhesin binding to carbohydrates on mucus [37]. This protein was later identified to be present in the outer membrane of *H. pylori* by proteomic analysis [38]. However, it has also been reported that HP-NAP is mainly localized in the cytosol of *H. pylori* [39]. This protein may be released upon autolysis. Because of its presence in the outer membrane, one might speculate that HP-NAP can be delivered by the outer membrane vesicles (OMVs) derived from *H. pylori*. Indeed, HP-NAP has been identified as a component of *H. pylori*-derived OMVs [40,41]. Because HP-NAP can cross the epithelial monolayers to simulate immune responses [31], the translocation of HP-NAP from the apical to the basolateral domain of the epithelial cells could also be mediated by OMVs. Once HP-NAP is released from the bacteria, it can directly interact with TLR2, a pattern recognition receptor, on neutrophils or monocytes to induce the secretion of cytokines [29,30]. This protein can also be sensed by a PTX-sensitive GPCR on neutrophils or monocytes to induce the production of ROS or mast cells to induce the secretion of IL-6 and histamine [22,31,32]. The unidentified PTX-sensitive GPCR may also participate in the secretion of IL-8 by neutrophils [29]. What this PTX-sensitive GPCR is and how the cellular signals transduced by the two receptors, TLR2 and the PTX-sensitive GPCR of HP-NAP, orchestrate HP-NAP-induced immune responses remain interesting questions to address.

## 3. HP-NAP as a Therapeutic Target for *H. pylori* Infection

HP-NAP acts as a pathogenic factor by activating a wide range of human leukocytes to induce gastric inflammation caused by *H. pylori* infection. The discovery of the inhibitory effect of arabinogalactan proteins (AGPs) extracted from Chios mastic gum (CMG), the natural resin of the plant *Pistacia lentiscus* var. *Chia*, on HP-NAP-induced adhesion of neutrophils to endothelial cells raises the possibility that HP-NAP could serve as a target for anti-inflammatory therapy in *H. pylori*-infected patients [42]. It was later reported that AGPs bound to specific membrane proteins from human neutrophils and inhibited HP-NAP-induced production of ROS by neutrophils [43]. Because only PTX-sensitive G proteins but not TLR2 are involved in HP-NAP-induced ROS production by neutrophils [29], AGP may exert its inhibitory effect on the GPCR of HP-NAP. Thus, pharmacological targeting of the receptors of HP-NAP could provide a potential therapeutic strategy for the treatment of *H. pylori*-induced gastric inflammation.

Among the two receptors of HP-NAP, TLR2 is known to play a vital role in regulating immunity during pathogen infection. This receptor has been considered as a potential therapeutic target for infectious diseases [44,45]. Drug candidates that antagonize TLR2 have been evaluated in animal studies and clinical trials for their anti-inflammation abilities [46]. These drug candidates might have potential applications in the treatment of *H. pylori*-induced gastric inflammation. As to the PTX-sensitive GPCR of HP-NAP, its identity is not clear. However, HP-NAP-induced ROS production by neutrophils is solely mediated by this GPCR [29]. It has been reported that the C-terminal region of HP-NAP stimulated ROS production by neutrophils [17]. The helices H3 (Leu69-Leu75) and H4 (Lys89-Leu114) and the linking coils (His63-Thr68; Thr76-Ser88) located at the surface of the C-terminal region of HP-NAP were suggested to be the critical regions in stimulating ROS production by human neutrophils [17]. Thus, the GPCR of HP-NAP may recognize the polypeptide fragment containing the residues from His63 to Leu114 of HP-NAP. It is also possible that this polypeptide fragment acts as an antagonist of the GPCR of HP-NAP. Whether this possibility is true needs to be investigated.

In addition to targeting the receptors of HP-NAP, direct targeting of HP-NAP might be a more straightforward strategy for the treatment of *H. pylori*-induced gastric inflammation. Due to the development of antibody drugs, it has become more popular to use soluble protein ligands as drug targets [47]. Recently, we reported that a commercially available antibody is able to detect the polypeptide fragment containing the residues from Arg77 to Glu116 of HP-NAP [48]. Whether this antibody can be repurposed into an antibody drug for the treatment of *H. pylori*-associated gastric diseases awaits further investigations.

**Figure 1:**
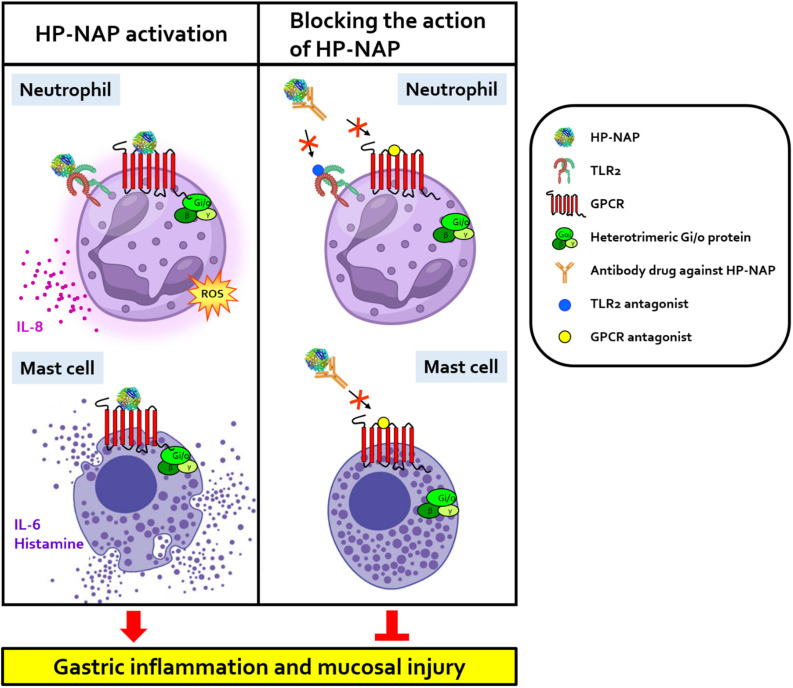
Schematic presentation of HP-NAP as a therapeutic target in the treatment of *H. pylori*-associated gastric diseases. The binding of HP-NAP to a PTX-sensitive GPCR induces the production of ROS by neutrophils and the secretion of IL-6 and histamine by mast cells. HP-NAP also induces neutrophils to release IL-8 via the activation of TLR2 and PTX-sensitive G proteins. Inhibition of the interactions between HP-NAP and its receptors by antibody drugs against HP-NAP and antagonists specific to the receptors of HP-NAP could relieve the gastric inflammation and mucosa injury caused by *H. pylori* infection. “↓” indicates induction; “⊥” indicates inhibition. GPCR, G protein-coupled receptor; HP-NAP, *H. pylori* neutrophil-activating protein; IL-6, interleukin-6; IL-8, interleukin-8; ROS, reactive oxygen species; TLR2, Toll-like receptor 2.

*H. pylori* has become increasingly difficult to be eradicated due to its high resistance to antibiotics [49,50]. HP-NAP is present in all *H. pylori* strains and is highly conserved among *H. pylori* strains [51]. Inhibition of the action of HP-NAP is a promising therapy for the treatment of *H. pylori*-associated diseases by reducing the gastric inflammation. Figure 1 shows how drugs designed to block the interactions between HP-NAP and its receptors are able to alleviate the gastric inflammation and mucosa injury caused by *H. pylori* infection. It has been reported that chronic gastritis caused by *H. pylori* infection increases the risk of developing gastric cancer [52,53]. A study in China showed that HP-NAP-specific antibody responses of *H. pylori*-infected patients with gastric cancer were significantly higher than those of *H. pylori*-infected patients with chronic gastritis [54]. The other study in Sweden showed that serum antibodies against HP-NAP are associated with the presence of non-cardia gastric adenocarcinoma [55]. These findings suggest that HP-NAP may be involved in the development of gastric carcinoma in *H. pylori*-infected patients. Thus, HP-NAP could act as a target for new drugs against gastric inflammation and maybe gastric cancer caused by *H. pylori* infection.

## 4. HP-NAP as Therapeutic Agents

HP-NAP acts as an immunogen to elicit antigenic responses or an immune modulator to regulate the activity of the immune system, especially T cell immunity. Due to its unique immune property, HP-NAP has potential applications in vaccine development, allergy treatment, and cancer immunotherapy. The potential clinical application of HP-NAP in allergy and cancer immunotherapy has also been reviewed elsewhere [56]. In this review, the application of HP-NAP as a therapeutic agent against various diseases in animal models is summarized in Table 1. HP-NAP may act as a variety of therapeutic agents, including a component of vaccines against *H. pylori* infection, a vaccine adjuvant, a drug candidate against allergic diseases, and an immunotherapeutic agent for cancer.

### 4.1. Vaccine Component

HP-NAP itself is an ideal vaccine component due to its strong antigenic response. The antigenicity of HP-NAP was reported in 2000, based on the finding that the serum from *H. pylori*-infected patients had a strong affinity to HP-NAP in an immunoblotting test [22]. In addition, oral immunization with recombinant HP-NAP together with a mucosal adjuvant provided a protective response in a mouse model [22]. The protective response resulted from immunization with HP-NAP was further shown by a study through delivering HP-NAP in a live *Lactococcus lactis* (*L. lactis*) vector in mice [65]. In this study, oral immunization of mice with *L. lactis* expressing HP-NAP showed elevated systemic and mucosal immune responses and reduced colonization of *H. pylori* [65]. The other study shows that oral priming and intramuscular boosting immunization of mice with the divalent vaccine containing HP-NAP and CagA enhanced both mucosal and systemic antibody responses to these two antigens of *H. pylori* [57]. In a beagle dog experimental model, intramuscular administration of the multivalent vaccine containing VacA, CagA, and HP-NAP with aluminum hydroxide as an adjuvant reduced bacterial colonization and gastritis in *H. pylori*-infected dogs [58]. These findings indicated that HP-NAP can be a component of both prophylactic and therapeutic vaccines against *H. pylori*. The same multivalent vaccine containing VacA, CagA, and HP-NAP with aluminum hydroxide generated specific antibody and T cell responses to all three antigens in healthy, *H. pylori*-negative volunteers in a phase 1 clinical study [59]. However, in a phase 1/2 clinical study reported 10 years later, this multivalent vaccine failed to provide additional protection against *H. pylori* infection in healthy volunteers after a challenge with a CagA-positive strain compared with a placebo [60]. The increased systemic humoral responses to the three *H. pylori* antigens induced by this vaccine were not sufficient to eradicate *H. pylori* [60]. Some research findings suggested that urease of *H. pylori* is an indispensable component in a multivalent vaccine against *H. pylori* infection [82]. In addition, an efficient mucosal adjuvant may be required for the development of a therapeutic vaccine against *H. pylori*. One research group has developed multivalent epitope-based vaccines containing HP-NAP and the epitopes of other *H. pylori* antigens and applied them in an *H. pylori*-infected Mongolian gerbil model. One of the vaccines consists of HP-NAP and the epitopes of urease, heat-shock protein 60 (HSP60), and *H. pylori* adhesin A (HpaA) with the mucosal adjuvant cholera toxin B subunit (CTB) [61]. The other one consists of HP-NAP and the epitopes of CagA, VacA, and urease with polysaccharide, a mucosal adjuvant [62]. Oral immunization of *H. pylori*-infected Mongolian gerbils with both multivalent epitope-based vaccines exhibited therapeutic effects, as shown by a marked reduction of gastritis and bacterial colonization [61,62]. The other research group developed a multivalent vaccine by formulating urease A and urease B, two subunits of urease, and HP-NAP with the mucosal adjuvant, a double-mutant heat-labile toxin (dmLT) from *Escherichia coli* [63]. Oral immunization of *H. pylori*-infected mice with this vaccine resolved gastritis but failed to fully eliminate the colonized bacteria, despite the induction of mucosal IgA responses and specific humoral immune responses [63]. Furthermore, the same multivalent vaccine adjuvanted with cyclic guanosine monophosphate-adenosine monophosphate (cyclic GMP-AMP, cGAMP) was able to protect mice against *H. pylori* infection with a much lower dosage through parenteral immunization, especially intranasal immunization, than oral immunization [64]. HP-NAP has been selected as a component to develop either prophylactic or therapeutic vaccines against *H. pylori* mainly due to its immunogenicity. The aforementioned vaccines were tested in different experimental models with some degree of success. However, further investigations on the mechanism by which *H. pylori* evades antibody-mediated attack from hosts are needed to enable the translation of these vaccines into effective human vaccines.

### 4.2. Vaccine Adjuvant

HP-NAP is not only considered as a main component of the vaccine against *H. pylori* but also a vaccine adjuvant due to its immunomodulatory property. HP-NAP has first been suggested as a Th1-inducing adjuvant by the finding that HP-NAP induced allergen-induced T cell lines to shift from a polarized Th2 phenotype to a predominant Th1 cell response [30]. In addition, the mode of action of HP-NAP as an adjuvant to enhance cellular immunity is shown by its ability to promote DC maturation with Th1-polarized responses and improve the uptake and presentation of antigen by mature DCs and the migration of DCs to draining lymph nodes [34]. The first evidence to support HP-NAP as an immunoadjuvant to enhance the immunogenicity of a poor immunogen was reported in a study using an attenuated measles virus expressing an HP-NAP-tagged antigen [83]. In this study, mice immunized with measles virus expressing recombinant human immunoglobulin lambda light chain only showed a low antibody response against the lambda light chain, while mice immunized with measles virus expressing chimeric lambda light chain with HP-NAP significantly increased the antibody response against the lambda light chain [83]. A recent study also reported that the immunogenicity of genetically-modified oncolytic vaccinia virus expressing the mimotope of disialoganglioside (GD2), a neuroblastoma-associated antigen, could be improved by the co-expression of the mimotope of GD2 together with HP-NAP [66]. These findings support a potential role of HP-NAP as an immunostimulatory adjuvant in DNA vaccines.

### 4.3. Drug Candidate against Allergic Diseases

The immunomodulatory property of HP-NAP not only makes HP-NAP a potential vaccine adjuvant but also makes it a potential drug candidate against allergic diseases. Due to its ability to drive a pro-allergic Th2 response toward a Th1 response, HP-NAP plays a protective role in several allergic diseases, including allergic asthma, atopic dermatitis, and food allergy. Such a protective role of HP-NAP was first reported for allergic asthma. In a mouse model of allergic asthma induced by ovalbumin (OVA), both systemic and mucosal administrations of HP-NAP inhibited eosinophil infiltration in lungs and Th2-mediated bronchial inflammation by reducing the OVA-induced production of Th2 cytokines, including IL-4, IL-5, and granulocyte-macrophage colony-stimulating factor (GM-CSF), in bronchoalveolar lavage (BAL) fluids and the level of IgE in serum and BAL fluids [67]. In addition, TLR2 is required for HP-NAP-mediated suppression of the level of Th2 cytokines in BAL fluids from mice with OVA-induced allergic asthma [67]. The other study suggests that HP-NAP could prevent allergic asthma through systemic and mucosal pre-administration [68]. In this study, the mice were pre-administered with HP-NAP via intraperitoneal injection or inhalation followed by sensitization and challenge with OVA to induce asthma [68]. Pre-administration with HP-NAP increased the levels of Th1 cytokine, IFN-γ, and the regulatory T cell (Treg) cytokine, IL-10, in BAL fluids and reduced the levels of Th2 cytokines, including IL-4 and IL-13, in BAL fluids, the level of IgE in serum, and the degree of eosinophils infiltration to the airway submucosa, indicating that HP-NAP prevents the development of asthma induced by OVA in mice [68]. A similar effect on the reduction of airway inflammation in the same mouse model of allergic asthma was achieved by oral administration of *Bacillus subtilis* (*B. subtilis*) spores with surface expression of HP-NAP fused to the CTB, a mucosal adjuvant inducing antigen tolerance [69] and the administration of the plasmid to express the fusion protein of HP-NAP and soluble IL-4 receptor (sIL-4R), a decoy receptor to bind IL-4 released by Th2 cells and eosinophils [70]. In both cases, the level of IFN-γ in BAL fluids was increased and the level of IL-4 in BAL fluids, the level of OVA-specific IgE in serum, and the degree of eosinophil infiltration in lungs were all reduced [69,70]. The level of IL-10 in BAL fluids and the number of CD4^+^CD25^+^Foxp3^+^ Tregs in splenocytes were increased in the mice, with oral administration of *B. subtilis* spores expressing HP-NAP fused to the CTB [69], whereas there was no significant difference in the RNA levels of Th17/Treg cytokines in the lung tissues of mice with administration of the plasmid expressing a fusion protein of HP-NAP and sIL-4R or the plasmid expression sIL-4R alone [70]. Nevertheless, all the findings from the studies using mice with OVA-induced allergic asthma support that HP-NAP could be a potential therapeutic agent in the prevention and treatment of allergic asthma.

In addition to amelioration of asthma, HP-NAP could ameliorate skin allergies such as atopic dermatitis and food allergies such as peanut allergy. One study, using a mice model with atopic dermatitis induced by oxazolone, shows that the treatment with HP-NAP via intraperitoneal injection alleviated the symptoms of atopic dermatitis by reducing the infiltration of lymphocytes and mast cells, the secretion of IgE and IL4, and the expression of inflammatory cytokines, including IL-1β, IL-5, IL-6, and TNF-α, in ear tissues of the animals [71]. In another study, oral administration of *B. subtilis* spores with surface expression of HP-NAP or CTB-conjugated HP-NAP in peanut-allergy-induced mice ameliorated anaphylactic reactions by increasing the levels of peanut-specific IgA in fecal and peanut-specific IgG2a in serum and by reducing the levels of histamine in plasma and peanut-specific IgE and IgG1 in serum [72]. The reduced allergic reactions to peanuts in these mice were also contributed by an increased secretion of IL-10 and IFN-γ and a decreased secretion of IL-4 and IL-5 by spleen cells [72]. In addition, the number of CD4^+^CD25^+^Foxp3^+^ Tregs was increased in mice with the administration of *B. subtilis* spores expressing HP-NAP or CTB-conjugated HP-NAP [72]. Pre-injection of the antibody against CD25, an IL-2 receptor α chain, suppressed the protective effect of HP-NAP on peanut allergy, indicating that HP-NAP-mediated suppression of peanut allergy is regulated by the activation of Tregs [72]. An earlier study applying protein dynamics simulations suggested that CD25 on Tregs could be the receptor of HP-NAP [84]. If this is the case, HP-NAP could directly activate Tregs to exert their immunosuppressive action on allergen-specific Th2 cells by binding to the CD25 receptor. However, further investigation is needed to test this hypothesis.

**Figure 2:**
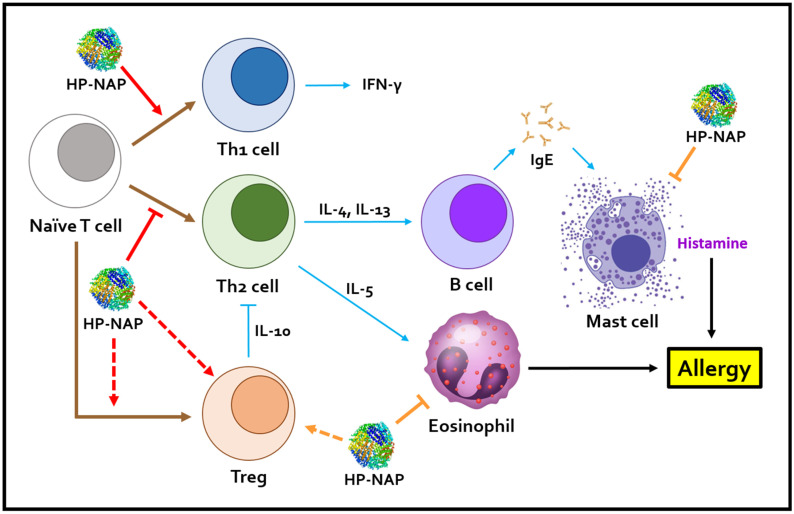
Schematic presentation of how HP-NAP reduces the allergy by shifting the immune response from a Th2 to a Th1 response. HP-NAP promotes the differentiation of naïve T cells into Th1 cells and suppresses the differentiation of naive T cells into Th2 cells, thus increasing the release of Th1 cytokines, such as IFN-γ, and reducing the release of Th2 cytokines, including IL-4, IL-5, and IL-13, respectively. The reduced Th2 cytokine production and reduced B cell-mediated immune response could lead to a reduction of histamine secretion by mast cells. The increased number of Tregs induced by HP-NAP could be due to the promotion of their differentiation or their infiltration into mucosa tissue. HP-NAP could promote the secretion of Treg cytokine, IL-10, leading to the suppression of activity of Th2 cells. HP-NAP also reduced the infiltration of eosinophils into airway mucosa tissues in allergic asthma and mast cells to the skin tissue in atopic dermatitis. “↓” indicates induction; “⊥” indicates inhibition; brown arrows indicate differentiation; red arrows indicate the stimulation of cell differentiation by HP-NAP; blue arrows indicate stimulation of cellular activity; orange arrows indicate the stimulation of cell infiltration by HP-NAP; dot arrows indicate the possible stimulation which has not been directly proved in research papers. HP-NAP, *H. pylori* neutrophil-activating protein; Th1, T-helper type 1; Th2, T-helper type 2; Treg, regulatory T cell; IL-4, interleukin-4; IL-5, interleukin-5; IL-10, interleukin-10; IL-13, interleukin-13; IFN-γ, interferon-γ; IgE, immunoglobulin E.

Collectively, HP-NAP can remodel the Th1/Th2 balance and relieve allergic symptoms in the aforementioned mouse models with allergic diseases, including allergic asthma, atopic dermatitis, and peanut allergy (Figure 2). Thus, HP-NAP is a potential therapeutic drug for Th2-mediated allergic diseases.

### 4.4. Immunotherapeutic Agent for Cancer

The immunomodulatory ability of HP-NAP to promote the Th1 immune response and CTL activity suggested that HP-NAP could be applied in cancer immunotherapy. HP-NAP as a therapeutic agent against tumors was first reported in a study using a mouse model of a bladder cancer implant. Administration of recombinant HP-NAP in mice reduced the growth of bladder cancer by triggering tumor necrosis and inducing a strong Th1 cytotoxic response both within tumors and local lymph nodes [73]. No such anti-tumor effect was observed in TLR2-knockout mice treated with HP-NAP, indicating that the TLR2-mediated signaling pathway is required for HP-NAP-induced responses [73]. Similar anti-tumor and immunomodulatory effects were also reported in mice bearing hepatoma or sarcoma tumors with the administration of recombinant HP-NAP fused with maltose binding protein (rMBP-NAP) [74]. Such effects induced by rMBP-NAP are also TLR2-dependent as shown by the fact that co-administration of TLR2-blocking antibody and rMBP-NAP inhibited the anti-tumor activity elicited by rMBP-NAP [74]. In addition to the in-situ tumor, rMBP-NAP was able to promote anti-tumor activity in a mouse model of melanoma with lung metastasis [75]. In peripheral blood mononuclear cells from patients with lung cancer, rMBP-NAP also induced the production of anti-tumor cytokines and promoted CTL activity [85]. Thus, recombinant HP-NAP, as a potent immune modulator, is a promising therapeutic agent for the treatment of various types of cancers.

To specifically deliver recombinant HP-NAP to the tissue of concern and provide controlled release therapy, chitosan nanoparticles were used as the carriers for recombinant HP-NAP and this nanoparticle-protein complex was applied in a mouse xenograft model with breast cancer. In the mice injected with chitosan nanoparticles loaded with recombinant HP-NAP, their survival rates and the rates of tumor shrinkage were higher than those in mice injected with recombinant HP-NAP alone [77]. The higher efficacy of chitosan nanoparticles loaded with recombinant HP-NAP against breast cancer in mice could be due to the reason that chitosan nanoparticles act as adjuvants to the main immunotherapy protein [86]. It is worth evaluating the efficacy of this therapeutic approach in other types of cancers.

HP-NAP is not only a potent immune modulator but also a promising immune adjuvant in cancer treatment. A study showed that recombinant HP-NAP promotes the maturation of DC-based vaccine loaded with lysates from melanoma cells and this vaccine was able to enhance the activation, proliferation, and cytotoxic response of melanoma tumor cell-specific T cells, supporting the role of HP-NAP as an adjuvant of a DC-based vaccine to enhance anti-melanoma responses [87]. Several studies have also explored the possibility of using HP-NAP as an immune modulator or an adjuvant in oncolytic virus-based cancer therapy in mouse models. The earliest study is the application of an attenuated measles virus strain engineered to express secretory HP-NAP in mouse xenograft models of metastatic breast cancer. Administration of oncolytic measles virus expressing secretory HP-NAP improved the survival of the mice with metastatic breast cancer and increase the level of pro-inflammatory cytokines, including IL-12/23, TNF-α, and IL-6, in pleural fluids of mice with pleural metastasis of breast cancer [78]. Another study shows that intratumoral administration of a replication-selective, infection-enhanced adenovirus expressing secretory HP-NAP in nude mice xenografted with neuroendocrine tumors prolonged their survival and reduced their tumor growth [79]. The treatment of mice with this oncolytic adenovirus expressing secretory HP-NAP induced neutrophil infiltration in the tumors and necrosis of the tumors [79]. The pro-inflammatory cytokines, IL-12/23, TNF-α, and MIP2-α, were also increased in the plasma of these treated mice [79]. The other study shows that concurrent expression of HP-NAP in oncolytic vaccinia virus enhanced its anti-tumor efficacy in mice with neuroblastoma as indicated by an increase in the survival rate and a reduction of the tumor size in mice with administration of the vaccinia virus co-expressing HP-NAP and the mimotope of GD2, a tumor-associated antigen (TAA) for neuroblastoma [66]. The serum level of anti-GD2 antibody in mice treated intratumorally with vaccinia virus co-expressing HP-NAP and the mimotope of GD2 was higher than that in mice treated intratumorally with vaccinia virus expressing the mimotope of GD2 alone [66], supporting the role of HP-NAP as an adjuvant to enhance the TAA-specific immune response.

Furthermore, HP-NAP, as an immunostimulator, can improve the potency of immune checkpoint inhibitor therapy and the efficacy of chimeric antigen receptor (CAR) T ccell therapy for solid tumors. In the case of immune checkpoint inhibitor therapy, the combination immunovirotherapy with anti-PD-1 antibody and oncolytic measle virus expressing secretory HP-NAP together with urokinase-type plasminogen activator receptor exerted an effective anti-tumor effect against glioblastoma in a mouse model [80]. The survival outcome of mice receiving combination immunovirotherapy was better than that of mice receiving therapy with anti-PD-1 antibody alone [80]. A higher infiltration of leukocytes, macrophages, CD4^+^ T cells, and CD8^+^ T cells into the brain was observed in mice receiving this combination immunovirotherapy [80]. Antibody depletion of CD8^+^ T cells led to a complete loss of the survival benefit of mice receiving combination immunovirotherapy, indicating that CD8^+^ T cell-mediated response is required for the therapeutic efficacy of this combination immunovirotherapy [80]. In terms of CAR T cell therapy, mouse CAR T cells expressing secretory HP-NAP led to better survival rates and slower growth of tumors in mice with subcutaneous lymphomas, neuroblastomas, pancreatic ductal adenocarcinomas, or colon carcinomas than the conventional mouse CAR T cells [81]. In mice treated with CAR T cells expressing secretory HP-NAP, higher levels of chemokines and Th1-related cytokines together with higher infiltration levels of neutrophils, M1 macrophages, cytotoxic natural killer cells, antigen-presenting DCs, and highly activated CD8^+^ T cells were detected in their tumor tissues [81]. The bystander T cell responses against solid tumors triggered by the secreted HP-NAP from CAR T cells could be mainly caused by the epitope spreading and infiltration of cytotoxic CD8^+^ T cells targeting TAAs other than the CAR-targeted antigen in the tumor microenvironment [81]. Thus, HP-NAP is a potential drug candidate for cancer immunotherapy due to its pluripotent property in activation and amplification of anti-tumor T cell responses.

HP-NAP also exerts its anti-tumor activity by modulating innate immune cells. In the zebrafish model of melanoma xenograft, treatment with HP-NAP not only promoted the survival of the fish but also reduced the growth and metastasis of melanoma cells [76]. The anti-tumor effect elicited by HP-NAP is mainly due to the recruitment of macrophages at the tumor sites and the polarization of recruited macrophages toward a pro-inflammatory, anti-tumor profile [76]. The expression of pro-inflammatory cytokines, IL-1β and IL-6, was upregulated in macrophages isolated from the fish treated with HP-NAP while the expression of the anti-inflammatory cytokine, IL-10, was downregulated in these macrophages [76]. Such anti-tumor activity of HP-NAP was lost in the zebrafish, whose macrophages were depleted [76], supporting that activated macrophages play a pivotal role in the anti-tumor activity of HP-NAP.

**Figure 3:**
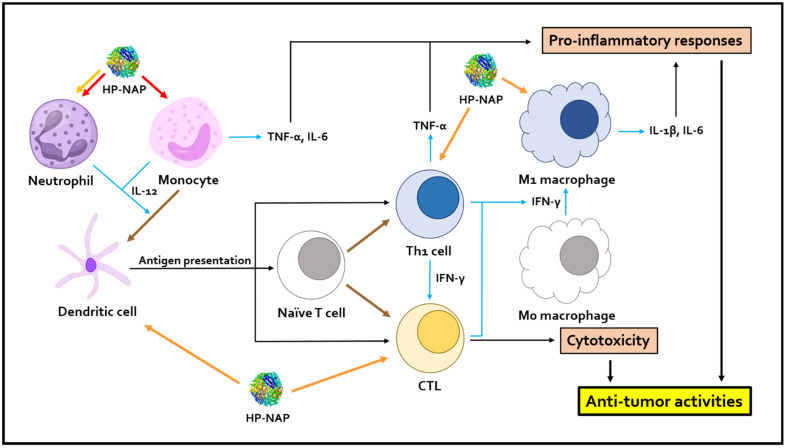
Schematic presentation of how HP-NAP promotes anti-tumor immune responses by inducing pro-inflammatory responses and CTL activity. HP-NAP directly activates neutrophils and monocytes to induce the secretion of IL-12. Activated monocytes also secrete TNF-α and IL-6 to promote anti-tumor inflammatory responses. The release of IL-12 could promote the differentiation of monocytes into dendritic cells (DCs). Mature dendritic cells promote the differentiation of naïve T cells into Th1 cells by antigen presentation, and further lead to the release of Th1 cytokines, such as IFN-γ and TNF-α, from Th1 cells. The polarization of M1 macrophages could be promoted by IFN-γ. M1 macrophages secrete IL-1β and IL-6 into tumor microenvironments and enhance inflammation responses. Antigen presentation, mediated by DCs, and Th1 cytokines could promote the differentiation and activation of CTLs to mediate anti-tumor immunity. HP-NAP also induces the infiltration of neutrophils, M1 macrophages, antigen-presenting DCs, Th1 cells, and CTLs into tumor microenvironments. “↓” indicates induction; red arrows indicate the direct stimulation of the cells by HP-NAP; orange arrows indicate the stimulation of cell infiltration by HP-NAP; blue arrows indicate stimulation of cellular activity; brown arrows indicate differentiation. HP-NAP, *H. pylori* neutrophil-activating protein; Th1, T-helper type 1; CTL, cytotoxic T lymphocyte; IL-1β, interleukin-1β; IL-6, interleukin-6; IL-12, interleukin-12; IFN-γ, interferon-γ; TNF-α, tumor necrosis factor-α.

Collectively, HP-NAP can be applied as a recombinant protein or expressed in the oncolytic virus, and even CAR T cells, as a secreted protein to serve as an immunotherapeutic agent against cancer. Figure 3 shows how HP-NAP induces the pro-inflammatory responses and CTL activity to promote anti-tumor immune responses. By acting as an immune modulator, an adjuvant, or an immune stimulator to activate both adaptive and innate immune cells, HP-NAP is a potential immunotherapeutic agent to improve the clinical outcome of cancer treatment.

## 5. Conclusions

HP-NAP was first identified as a bacterial factor of *H. pylori* to stimulate neutrophil responses. Since *H. pylori* infection led to a massive infiltration of neutrophils and mononuclear cells into the gastric mucosa [2,3], it is thought that HP-NAP acts as a virulence factor by the recruitment of these innate immune cells to the site of *H. pylori* infection to cause the gastric inflammation. Many efforts have then been focused on the identification of its receptors and investigation of the actions of HP-NAP on other immune cells, including both innate and adaptive immune cells. The PTX-sensitive GPCR of HP-NAP seems to mainly participate in the innate immune responses induced by HP-NAP, whereas TLR2 is involved in both innate and Th1-adaptive immune responses induced by HP-NAP. The inflammatory immune responses elicited by HP-NAP through these two receptors in both innate and adaptive immune cells contribute to the gastric damage caused by *H. pylori* infection, further supporting the role of HP-NAP in the pathogenesis of *H. pylori*. The development of new drugs by targeting the two receptors of HP-NAP or HP-NAP itself could provide an alternative treatment for *H. pylori*-associated gastric inflammation. However, more remains to be investigated on the identification of the GPCR of HP-NAP and how HP-NAP interacts with its two receptors to make this treatment strategy feasible for alleviating gastric inflammation caused by *H. pylori* infection.

As more is known about the immune properties of HP-NAP, it is much clearer how HP-NAP can be applied for therapeutic purposes. HP-NAP is an antigen and acts as an adjuvant to trigger and enhance immune responses. As a TLR2 agonist, HP-NAP can also drive a pro-allergic Th2 response toward a Th1 response or induce the pro-inflammatory Th1 responses and CTL responses against cancer. Such unique immune properties of HP-NAP make it a promising therapeutic agent with a variety of roles, such as a vaccine against *H. pylori*, a drug against allergic diseases, and an immunotherapeutic agent for cancer treatment. The application of HP-NAP as a therapeutic agent in the field of cancer immunotherapy is especially attractive. The ability of HP-NAP to stimulate Th1 immunity can boost the immune responses against certain types of cancers which respond poorly to current immunotherapies. The findings from the studies using combination therapies of HP-NAP together with an anit-PD1 immune checkpoint inhibitor or with CAR T cells in mouse models have already shown us that HP-NAP could enhance the immune responses against tumor cells and increase the infiltration of immune cells into tumor microenvironments. A combination of immunotherapy with HP-NAP could trigger stronger immune responses against the tumors which were supposed to be tolerant or resistant to single immunotherapy. The combination therapy of HP-NAP with other immunotherapies used in cancer treatment not only can improve the effectiveness of the approved therapy against certain types of cancers but might also expand the range of cancers it can treat. Although the therapeutic application of HP-NAP has been extensively investigated using animal models, successful translation of the results from animal models to humans is essential to enable HP-NAP to become a therapeutic medicine.

## Figures and Tables

**Table 1:** Potential applications of HP-NAP as therapeutic agent in vaccine development, allergy treatment, and cancer immunotherapy.

Application	Disease	Form of the Therapeutic Agent	Mode of Action	References
**Prophylactic and therapeutic vaccines**	*H. pylori*-infection	Recombinant protein	Antigenicity	[22,57,58,59,60,61,62,63,64]
		*Lactococcus lactis* expressing HP-NAP		[65]
**Vaccine adjuvant**	Neuroblastoma	DNA cancer vaccine	Stimulation of Th1-polarizing capacity of dendritic cells Enhancement of Th1-polarized responses Enhancement of the immunogenicity of antigens	[66]
**Drug candidate against allergic diseases**	Allergic asthma	Recombinant protein	Suppression of pro-allergic Th2 responses Promotion of Th1 immune responses	[67,68]
		DNA plasmid expressing HP-NAP		[69]
		*Bacillus subtilis* (*B. subtilis*) spores with surface expression of HP-NAP		[70]
	Atopic dermatitis	Recombinant protein		[71]
	Peanut allergy	*B. subtilis* spores with surface expression of HP-NAP		[72]
**Immunotherapeutic agent against cancer**	Bladder cancer	Recombinant protein	Promotion of Th1 immune responses Promotion of cytotoxic T lymphocyte responses Promotion of M1 macrophage polarization	[73]
	Hepatoma tumor			[74]
	Sarcoma tumor			[74]
	Melanoma			[75,76]
	Breast cancer			[77]
	Breast cancer	Oncolytic virus expressing HP-NAP		[78]
	Neuroendocrine tumor			[66,79]
	Glioblastoma			[80]
	Lymphoma	Chimeric antigen receptor T cell expressing HP-NAP		[81]
	Neuroblastoma			[81]
	Pancreatic ductal adenocarcinoma			[81]
	Colon carcinoma			[81]
